# Congenital solitary neck ulcer as a presentation of Langerhans cell histiocytosis

**DOI:** 10.1093/skinhd/vzaf050

**Published:** 2025-08-25

**Authors:** Bahareh Abtahi-Naeini, Maryam Derakhshan, Ali Emamjomeh, Azam Ghehsareh Ardestani, Somayeh-Sadat Momenzadeh, Mahsa Pourmahdi-Boroujeni

**Affiliations:** Pediatric Dermatology Division of Department of Pediatrics, Imam Hossein Children’s Hospital, Isfahan University of Medical Sciences, Isfahan, Iran; Skin Diseases and Leishmaniasis Research Center, Isfahan University of Medical Sciences, Isfahan, Iran; Department of Pathology, Isfahan University of Medical Sciences, Isfahan, Iran; Student Research Committee, Isfahan University of Medical Sciences, Isfahan, Iran; Child Growth and Development Research Center, Research Institute for Primordial Prevention of Non-Communicable Disease, Isfahan University of Medical Sciences, Isfahan, Iran; Department of Pediatrics, Imam Hossein Children’s Hospital, Isfahan University of Medical Sciences, Isfahan, Iran; Student Research Committee, Isfahan University of Medical Sciences, Isfahan, Iran

## Abstract

Langerhans cell histiocytosis (LCH) is a neoplasm originating from immature haematopoietic myeloid precursor cells. It may uncommonly present with congenital ulcers. We report a case of a neonate admitted with a fever and a purulent congenital ulcer at the base of the neck, who, in the investigations, was diagnosed with LCH. This case highlights the importance of considering LCH in persistent, treatment-resistant cutaneous lesions in neonates and the necessity for biopsy and continuous monitoring.

## Teaser

Langerhans cell histiocytosis (LCH) is a neoplasm originating from immature haematopoietic myeloid precursor cells. It may uncommonly present with congenital ulcers. We report a case of a neonate admitted with a fever and a purulent congenital ulcer at the base of the neck, which, in the workups, was diagnosed with LCH. This case highlights the importance of considering LCH in persistent, treatment-resistant cutaneous lesions in neonates and the necessity for biopsy and continuous monitoring.

What is already known about this topic?

- Langerhans cell histiocytosis (LCH) is a rare neoplasm and commonly involves multisystem manifestations, including cutaneous lesions.
- Isolated skin involvement in LCH, particularly presenting as a solitary ulcerated lesion, is extremely rare, with fewer than 50 reported cases worldwide.

What does this study add?

- This study reports a unique case of congenital LCH presenting as a solitary ulcerated skin lesion in a neonate, challenging previous categorizations and highlighting the potential for spontaneous regression in such cases.
- The findings emphasize the importance of long-term follow-up for isolated cutaneous LCH to monitor for any progression to multisystem involvement despite a generally favourable prognosis.

Langerhans cell histiocytosis (LCH) represents a neoplasm originating from immature haematopoietic myeloid precursor cells, often associated with the *BRAF*-V600E mutation. It is estimated to affect 4–5 per 1 million children younger than 15 years of age.^1,2^ Historically, LCH was classified into distinct entities based on clinical presentation and the extent of systemic involvement: (i) Hand–Schüller–Christian, (ii) eosinophilic granuloma, (iii) Letterer–Siwe and (iv) Hashimoto–Pritzker.^2–4^ However, cumulative clinical experience has revealed that this classification is insufficient due to the considerable overlap in manifestations.^2^

Isolated skin involvement occurs in only 2% of cases. The congenital form, known as Hashimoto–Pritzker disease, is characterized by congenital self-healing multiple red–purple or brown papules.^2–4^ Nevertheless, only-skin involvement congenital LCH presenting with isolated cutaneous lesion is another clinical form that has made the previous system of categorization obsolete.^2^ Here, we report a male neonate with a congenital neck ulcer who was diagnosed with LCH following further investigations.

## Case report

**Figure 1:**
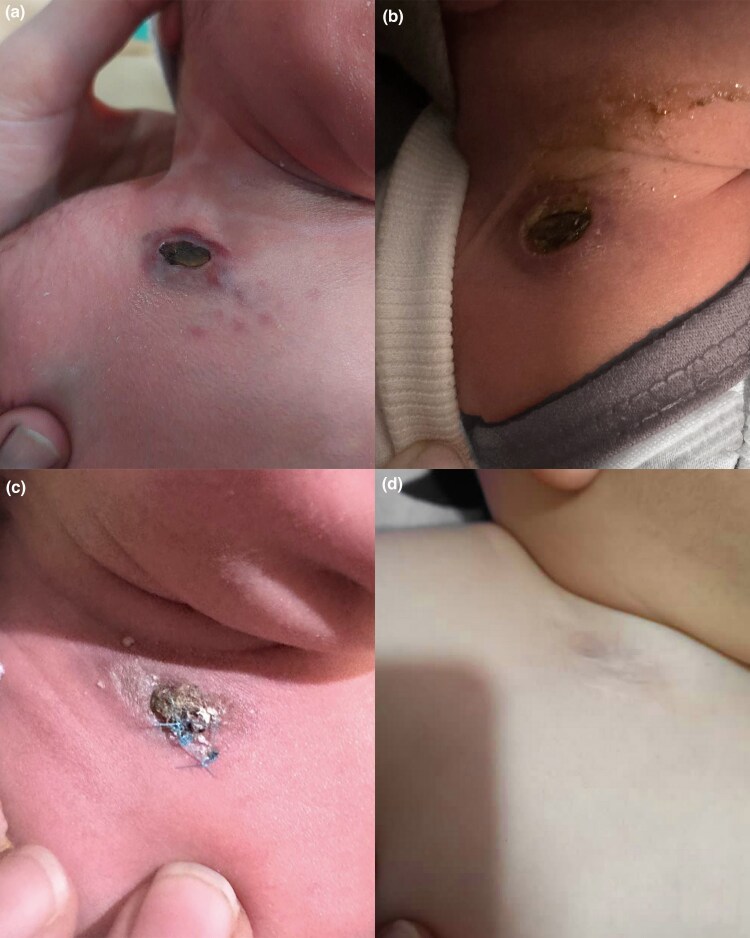
Congenital Langerhans cell histiocytosis in a male neonate presenting with a solitary ulcerated lesion: (a) a single 1 × 1 cm ulcer on the base of the neck with a necrotic base and erythematous borders at birth; (b) the 1 × 2 cm ulcer with a necrotic base and erythematous, indurated borders and purulent discharge and crusts at admission; (c) considering the unresponsive status of the ulcer after receiving antibiotics, biopsy was obtained; and (d) the lesion resolved spontaneously and left an atrophic scar.

A 2-week-old neonate was admitted to the hospital with a 2-day history of fever and a solitary purulent ulcer at the base of the neck. Since birth, he had an ulcer in the same region, initially suspected to be an ulcerated congenital haemangioma, and was treated with beta-blockers (Figure 1a). However, the lesion did not exhibit typical characteristics of haemangiomas or other vascular lesions. Additionally, topical antibiotics were prescribed, but after 14 days, there was no improvement, and the ulcer showed a heavy purulent discharge. Consequently, the neonate was hospitalized for sepsis work-up and further evaluation of the ulcer’s origin.

The patient was born full-term via caesarean section, weighing 3.3 kg, with an Apgar score of 9/10. Due to the presence of the ulcer and concerns about possible immune deficiency, he did not receive the Bacillus Calmette–Guérin (BCG) vaccination at birth. His family history was unremarkable.

The dermatological examination revealed an ulcer in the supraclavicular region, approximately 1 × 2 cm in size, with a necrotic base and erythematous, indurated borders (Figure 1b). Suspecting an infected ulcer, the patient was treated with clindamycin and ceftriaxone. Although antibiotic administration significantly reduced the purulent discharge, there was only a slight reduction in the size and induration of the ulcer. Further evaluation for other infectious aetiologies, including *Mycobacterium* and *Herpesviridae* infections, returned negative results.

Subsequently, a skin biopsy of the lesion was performed, to investigate various differential diagnoses, including ulcerated deep haemangioma, ulcerated mastocytoma, congenital leukaemia cutis, congenital soft tissue tumour and congenital histiocytoma (Figure 1c).

**Figure 2:**
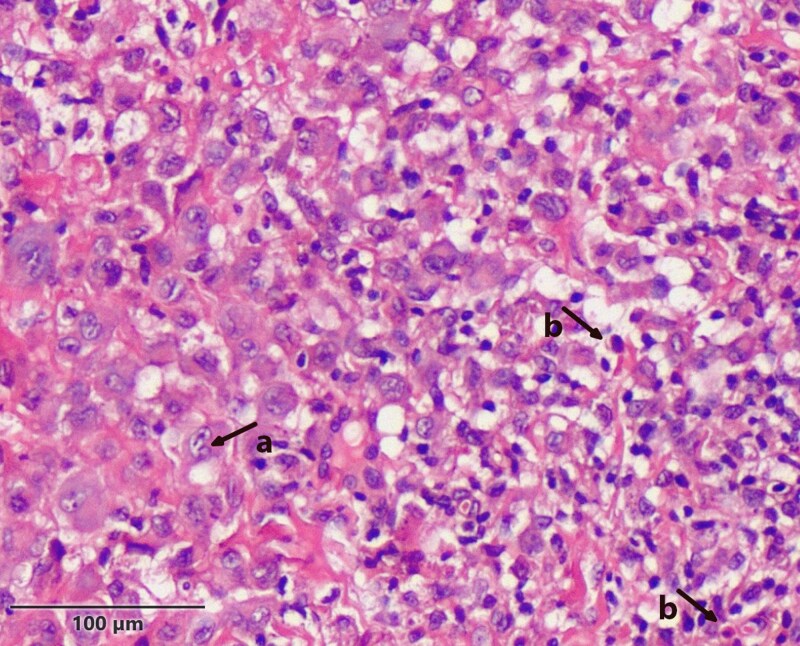
Congenital Langerhans cell histiocytosis: skin biopsy revealed a dense perivascular and interstitial infiltration of large histocytes (a, arrow) and eosinophils (b, arrow) in the dermis. Histiocytes showed eosinophilic cytoplasm and reniform nuclei (haematoxylin and eosin; × 40 magnification; scale bar = 100 μm).

**Figure 3:**
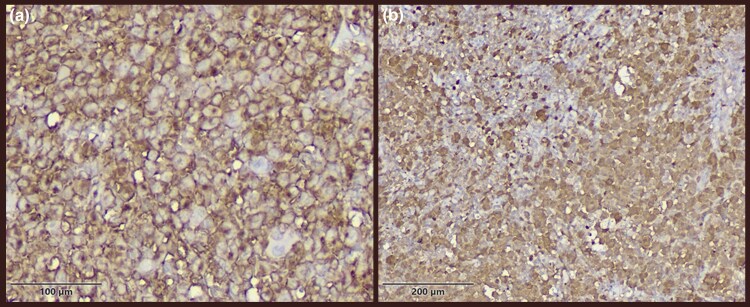
Immunohistochemistry staining of congenital Langerhans cell histiocytosis: (a) strong positive membranous immunostaining for CD1a (× 40 magnification; scale bar = 100 μm); (b) strong positive nuclear and cytoplasmic immunostaining for S100 ( × 40 magnification; scale bar = 200 μm).

Histopathological evaluation of the skin biopsy revealed an atrophic epidermis and dense dermal infiltration of cells with irregular to folded nuclei with prominent nuclear grooves, vesicular chromatin, distinct nucleoli and moderate amounts of pale eosinophilic cytoplasm. Frequent admixed eosinophils were observed. The histiocytic infiltration extended into the deep dermis and subcutaneous fat (Figure 2). Immunohistochemistry studies demonstrated strong positivity for CD1a and positive expression for CD68 and S100, while CD31 and CD34 yielded negative results (Figure 3). Hence, the diagnosis of LCH was made.

Evaluation for disseminated disease, including a radiographic skeletal survey, chest radiograph, complete blood count with differential and hepatic function panel, revealed no abnormalities. Genetic analysis, including *BRAF* mutation testing, was not performed. The lesion resolved spontaneously, leaving an atrophic scar after 6 months. Follow-up evaluations by paediatric hematology–oncology at 3 and 6 months of age showed no signs of recurrence or dissemination of LCH (Figure 1d).

## Discussion

Diagnosing cutaneous manifestations of LCH can be challenging as they are often identified during secondary re-­evaluation of treatment-resistant lesions by performing biopsy and immunohistochemistry staining.^1^ Isolated cutaneous involvement in LCH with a single lesion is rare, with fewer than 50 reported cases worldwide. The solitary congenital cutaneous lesion is also known as histiocytoma.^2,3^ It can present as a painless papule or nodule with or without ulceration or an overlying scale crust.^4–6^

Ulceration is an uncommon presentation, with all reported cases compiled and summarized in Table 1. A comprehensive search of PubMed was conducted to identify English-language reports of congenital LCH with a solitary ulcerated lesion, and these cases are outlined in Table 1.^3–20^

**Table 1:** Cases of congenital Langerhans cell histiocytosis with solitary ulcerated lesion

Case no.	Author country/year	Gender	Location/size	Immunohistochemistry	Regression time	Prognosis	Other
1	Berger,^7^ USA, 1986	F	Right temporal/1 cm	S100+	18 weeks	NM	Electron microscopy: BG+
2		M	Dorsum of right foot/1 cm	0KT6+, 0KT4–	9 weeks	NM	Electron microscopy: BG+ , complicated prenatal period with maternal recurrent UTI
3		M	Hypothenar of right hand/NM	NM	NM	5-year F/U with no recurrence	Electron microscopy: BG+
4		M	Left inguinal/1 × 2 cm	S100+	12 months	1-year F/U with no recurrence	
5	Divaris,^10^ Canada, 1991	M	Scalp/1 cm	S100+	11 days	NM	Electron microscopy: BG+
6	Chun,^9^ Korea, 1992	F	Centre of her chin/papule-size	S100+	NM	NM	Electron microscopy: BG + , term neonate
7	Bernstein,^8^ USA, 1993	M	Medial of left thigh/1 cm	S100+, CD45–	3 months	6-month F/U with no recurrence	Electron microscopy: BG + , term neonate
8	Kirkland,^11^ England, 1996	M	Sole of the foot/nodule size	S100+	Excision biopsy	3-month F/U with no recurrence	Post-term neonate
9	Zunino-Goutorbe,^12^ France, 2008	M	Extremity of left eyebrow/5 mm	CD1a+, S100+	1.5 month	2-year F/U with no recurrence	Term neonate
10		M	Upper lip/1 cm	CD1a+, S100+	3 months	2-year F/U with no recurrence	Term neonate
11		F	Left back/1 cm	S100+, OKT6+	4 months	6-month F/U with no recurrence	Electron microscopy: BG + , term neonate
12		M	Right arm/1 cm	CD1a+, S100+	1 month	4-year F/U with no recurrence	Electron microscopy: BG + , term neonate
13		M	Left arm/1 cm	NM	1 month	2-year F/U with no recurrence	Electron microscopy: BG + , term neonate
14		F	Left flank/8 mm	CD1a+, S100+	2 months	2-year F/U with no recurrence	
15	Zwerdling,^19^ USA, 2009	M	Lateral of the right foot/13 mm	CD1a+, CD68+, S100+, CD207+	Excision biopsy	26-month F/U with no recurrence	Possibility of trauma during an amniocentesis procedure
16	Riva,^14^ Italy, 2009	M	Back/2 × 1.5 cm	S100+, CD68+, CD207+	4 months	15-month F/U with no recurrence	Term neonate
17		M	Medial of the right thigh/NM	S100+, CD68+, CD207+	4 months	10-month F/U with no recurrence	Term neonate
18	Wheller,^6^ Australia, 2013	M	Left posterior shoulder/5 mm	CD1a+, CD68+	5 months	NM	The mother had Crohn disease and consumed azathioprine and prednisolone during pregnancy
19	Yurkovich,^17^ Canada, 2013	F	Right hip/1.5 cm	CD1a+, S100+	NM	2-year F/U with no recurrence	
20		F	Right flank/1.8 cm	CD1a+, S100+	NM	2-year F/U with no recurrence	
21	Zanuncio,^18^ Brazil, 2013	M	Perianal/1 × 1.5 cm	CD1a+, S100+	2.5 months	NM	Term neonate
22	Uber,^15^ Brazil 2014	M	Superior back/NM	CD1a+, S100+	4 months	NM	
23	Lee,^13^ USA, 2014	F	Left posterior shoulder/8 mm	CD1a+	8 weeks	NM	The mother had a history of smoking, depression, bipolar disorder, lithium use, intrauterine growth restriction, term neonate
24	Udkoff,^16^ USA, 2018	F	Left-side scalp/2 cm	CD1a+, S100+	NM	NM	
25	McKenzie,^5^ USA, 2019	M	Posterior left thigh/6 mm	CD1a+	3 weeks	F/U NM, self-healing course	Genetic workups: V600E–
26	Ungari,^20^ Italy, 2021	F	Right arm/1 × 1.2 cm	CD1a+, S100+, CD207+, CD14+, CD45RB/LCA+, CD68R, PG-M1+, Ki-67+	4 months	F/U NM, self-healing course	Genetic workups: V600E–, term neonate
27	Lima,^4^ Brazil, 2022	M	Left leg/1.2 cm	CD1a+, S100+, CD207+, Ki67+	1 month	8-month F/U with no recurrence	*In vitro* fertilization, term neonate
28	Gomes,^3^ Brazil, 2023	M	Left scapular region/8 mm	CD1a+, S100+, CD207+, CD45+, CD68–, CD34–	NM	6-month F/U with no recurrence	Term neonate
29	Our case, Iran, 2024	M	Supraclavicular region/1 × 1 cm	CD1a+, CD68+, S100+, CD31–, CD34–	6 months	5-month F/U with no recurrence	Term neonate

BG, Birbeck granule; F, female; F/U, follow-up; M, male; NM, not mentioned; UTI, urinary tract infection.

The Hashimoto-Pritzker form is known for its excellent prognosis and has been termed congenital self-limited LCH. Isolated skin involvement is suggested to represent a sign of a good prognosis.^5,20^ Nevertheless, even cases with only skin involvement may progress to a multisystem disease requiring systemic treatment. Therefore, studies emphasize the importance of long-term follow-up (at least 2 years) at regular intervals due to the potential for recurrence and systemic involvement.^3^ Such a consequence has been reported in patients with multiple cutaneous lesions. Among the 29 cases outlined in Table 1 with LCH characterized by isolated, solitary ulcerated lesions, nearly all experienced spontaneous regression within 4–18 weeks without further complications.^1,5,6,17,20,21^

Surgical excision is often the preferred treatment for isolated skin lesions, although overtreatment should be avoided due to the benign course of the condition.^22^ As Table 1 shows, except for two patients with excision biopsy, the other patients were followed and achieved self-remission.

The need for diagnostic evaluation and continuous monitoring in cases of isolated skin involvement in LCH is still debatable, with no definitive findings shedding light on the disease’s progression. However, our case and previous ones assert that a solitary skin lesion with ulceration in a term neonate typically carries a favourable prognosis.^3,4,20^ In treatment-resistant lesions, biopsy and investigations should not be delayed. Although solitary cutaneous lesion presentation, with or without ulceration, is rare in LCH dermatologists must consider it as a possibility.

In conclusion, LCH presenting as a congenital solitary ulcer typically follows a benign course, self-resolving within 4–18 weeks without progressing to systemic involvement. However, these lesions require careful monitoring and diagnostic evaluation, with long-term follow-up to ensure no recurrence or systemic spread.

## Data Availability

The data underlying this article will be shared on reasonable request to the corresponding author.
